# Notification of changes in taxonomic opinion previously published outside the IJSEM. List of Changes in Taxonomic Opinion no. 44

**DOI:** 10.1099/ijsem.0.007140

**Published:** 2026-07-31

**Authors:** Aharon Oren, Markus Göker

**Affiliations:** 1 The Institute of Life Sciences, The Hebrew University of Jerusalem, The Edmond J. Safra Campus, 9190401 Jerusalem, Israel; 2 Leibniz Institute DSMZ – German Collection of Microorganisms and Cell Cultures, Inhoffenstrasse 7B, 38124 Braunschweig, Germany

The International Code of Nomenclature of Prokaryotes (ICNP) [1] deals with the nomenclature of prokaryotes. This may include existing names (the Approved Lists of Bacterial Names), as well as new names and new combinations. In this sense, the Code is also dealing indirectly with taxonomic opinions. However, as with most codes of nomenclature, there are no mechanisms for formally recording taxonomic opinions that do not involve the creation of new names or new combinations. In particular, it would be desirable for taxonomic opinions resulting from the creation of synonyms or emended descriptions to be made widely available to the public. In 2004, the Editorial Board of the *International Journal of Systematic and Evolutionary Microbiology* (IJSEM) agreed unanimously that it was desirable to cover such changes in taxonomic opinions (i.e. the creation of synonyms or the emendation of circumscriptions) previously published outside the IJSEM and to introduce a List of Changes in Taxonomic Opinion [2]. Scientists wishing to have changes in taxonomic opinion included in future lists should send a PDF file of the relevant publication to the IJSEM Editorial Office or to the Lists Editors.

It must be stressed that the date of proposed taxonomic changes is the date of the original publication, not the date of publication of the list. Taxonomic opinions included in the List of Changes in Taxonomic Opinion do not affect the status of a name under the International Code of Nomenclature of Prokaryotes [2] and can neither be considered as approved nor as disapproved by the International Committee on Systematics of Prokaryotes. The names that are to be used are those that are the ‘correct names’ (in the sense of Principle 6 of the ICNP) in the opinion of the microbiologist, with a given circumscription, position and rank. A particular name, circumscription, position and rank do not have to be adopted in all circumstances. Consequently, the List of Changes in Taxonomic Opinion must be considered as a service to microbiology, and it has no ‘official character’, other than providing a centralized point for registering/indexing such changes in a way that makes them easily accessible to the scientific community.

The List Editors reserve the right not to include in the list proposed emendations that are, in our opinion, unimportant and do not significantly alter the original description of the taxon. This includes addition of new members to the taxon, reclassification of members in a different taxon, addition of genome sequence data and other changes that marginally change the taxon circumscription [3]. Conversely, the mere inclusion of an emendation in a List of Changes in Taxonomic Opinion does not indicate that the List Editors regard this emendation as taxonomically relevant.

**Table:** 

Name/authors	Proposed as	Reference
*Actinomadura kijaniata* Horan and Brodsky 1982 emend. Lee *et al*. 2025, 9*	emend.	[4]
*Actinomadura namibiensis* Wink *et al*. 2003 pro synon. *Actinomadura kijaniata* Horan and Brodsky 1982	synon.	[4]
*Alicyclobacillus Wisotzkey et al.* 1992 emend. Sinnighe Damsté *et al.* 2025, 16*	emend.	[5]
*Alicyclobacillus tengchongensis* Kim *et al*. 2016 pro synon. *Alicyclobacillus tolerans* Karavaiko *et al*. 2005^†^	synon.	[5]
*Alicyclobacillus montanus* López *et al*. 2018 pro synon. *Alicyclobacillus tolerans* Karavaiko *et al*. 2005^†^	synon.	[5]
*Alteromonadaceae* Ivanova and Mikhailov 2001 emend. Flores-Félix *et al*. 2025, 2*	emend.	[6]
*Antarctobacter* Labrenz *et al*. 1998 emend. Xu *et al*. 2025, 11*	emend.	[7]
*Antrihabitans* Lee *et al*. 2020 emend. Lee *et al*. 2026, 10*	emend.	[8]
*Celerinatantimonadaceae* Cramer *et al*. 2011 emend. Flores-Félix *et al*. 2025, 3*	emend.	[6]
*Defluviimonas salinarum* Lee *et al*. 2025 emend. Song *et al*. 2026, 7*^‡^	emend.	[9]
*Endozoicomonadaceae* Bartz *et al*. 2018 emend. Flores-Félix *et al*. 2025, 2*	emend.	[6]
*Frateuria* Swings *et al*. 1980 emend. Bansal *et al*. 2023, 462	emend.	[10]
*Gulbenkiania indica* Jyoti *et al*. 2010 pro synon. *Gulbenkiania mobilis* Vaz-Moreira *et al*. 2007	synon.	[11]
*Halomonadaceae* Franzmann *et al*. 1989 emend. Flores-Félix *et al*. 2025, 2*	emend.	[6]
*Ketobacteraceae* Chuvochina *et al*. 2024 emend. Flores-Félix *et al*. 2025, 2*	emend.	[6]
*Marinobacteraceae* Liao *et al*. 2021 emend. Flores-Félix *et al*. 2025, 2*	emend.	[6]
*Marinomonadaceae* Chuvochina *et al*. 2024 emend. Flores-Félix *et al*. 2025, 2*	emend.	[6]
*Methylophaga aminisulfidivorans* Kim *et al*. 2007 pro synon. *Methylophaga thalassica* Janvier *et al*. 1985	synon.	[12]
*Methylophaga thalassica* Janvier *et al*. 1985 emend. Bayburt *et al*. 2026, 13*	emend.	[12]
Micromonospora veneta Kaewkla *et al*. 2022 pro synon. *Micromonospora coerulea* Jensen 1932 (Approved Lists 1980)	synon.	[13]
*Moraxellaceae* Rossau *et al*. 1991 emend. Flores-Félix *et al*. 2025, 2*	emend.	[6]
*Natronospirillaceae* Kevbrin *et al*. 2020 emend. Flores-Félix *et al*. 2025, 3*	emend.	[6]
*Niabella* Kim *et al*. 2007 emend. Dai *et al*. 2011 emend. Kim *et al*. 2025, 11*	emend.	[14]
*Oceanospirillaceae* Garrity *et al*. 2005 emend. Flores-Félix *et al*. 2025, 2*	emend.	[6]
*Oxalobacteraceae* Garrity *et al*. 2006 emend. Flores-Félix *et al*. 2025, 3*	emend.	[6]
*Planctomicrobium* Kulichevskaya *et al*. 2015 emend. Kallscheuer *et al*. 2026, 9*	emend.	[15]
*Pseudomonadaceae* Winslow *et al*. 1917 (Approved Lists 1980) emend. Flores-Félix *et al*. 2025, 2*	emend.	[6]
*Pseudomonadales* Orla-Jensen 1921 (Approved Lists 1980) emend. Flores-Félix *et al*. 2025, 2*	emend.	[6]
*Pseudomonas* Migula 1894 (Approved Lists 1980) emend. Flores-Félix *et al*. 2025, 2*	emend.	[6]
*Pseudonocardia saturnea* (Hirsch 1960) Warwick *et al*. 1994 pro synon. *Pseudonocardia autotrophica* (Takamiya and Tubaki 1956) Warwick *et al*. 1994	synon.	[16]
Qipengyuania Feng *et al*. 2015 emend. Lee *et al*. 2025, 10*^§^	emend.	[17]
*Robertmurraya yapensis* Hitch *et al*. 2025 pro synon. *Bacillus yapensis* Xu *et al*. 2025	synon.	[16]
*Shewanellaceae* Ivanova *et al*. 2004 emend. Flores-Félix *et al*. 2025, 3*	emend.	[6]
*Sphaerotilus* Kützing 1833 (Approved Lists 1980) emend. Smolyakov *et al*. 2025, 8*	emend.	[18]
*Sphingobium baderi* Kaur *et al*. 2013 pro synon. *Sphingobium wenxiniae* Wang *et al*. 2011	synon.	[19]
*Thermoanaerobacter brockii* (Zeikus *et al*. 1983) Lee *et al*. 1993 emend. Bouras *et al*. 2026, 7*	emend.	[20]
*Thermoanaerobacter ethanolicus* subsp. *ethanolicus* (Wiegel and Ljungdahl 1982) Bing 2026 pro synon. *Thermoanaerobacter thermohydrosulfuricus* (Klaushofer and Parkkinen 1965) Lee *et al*. 1993	synon.	[20]
*Thermoanaerobacter italicus* Kozianowski *et al*. 1998 pro synon. *Thermoanaerobacter thermocopriae* (Jin *et al*. 1988) Collins *et al*. 1994	synon.	[20]
*Thermoanaerobacter mathranii* Larsen *et al*. 1998 pro synon. *Thermoanaerobacter thermocopriae* (Jin *et al*. 1988) Collins *et al*. 1994	synon.	[20]
*Thermoanaerobacter pentosaceus* Tomás *et al*. 2013 pro synon. *Thermoanaerobacter thermocopriae* (Jin *et al*. 1988) Collins *et al*. 1994	synon.	[20]
*Thermoanaerobacter pseudethanolicus* Onyenwoke *et al*. 2007 pro synon. *Thermoanaerobacter brockii* (Zeikus *et al*. 1983) Lee *et al*. 1993	synon.	[20]
*Thermoanaerobacter thermocopriae* (Jin *et al*. 1988) Collins *et al*. 1994 emend. Bouras *et al*. 2026, 7*	emend.	[20]
*Thermoanaerobacter thermohydrosulfuricus* (Klaushofer and Parkkinen 1965) Lee *et al*. 1993 emend. Bouras *et al*. 2026, 6*	emend.	[20]
*Thermoanaerobacter* Wiegel and Ljungdahl 1982 emend. Bouras *et al*. 2026, 6*^¶^	emend.	[20]
*Thermodesulfobium narugense* Mori *et al*. 2004 emend. Maltseva *et al*. 2026, 11*	emend.	[21]
*Tundrisphaera* Kulichevskaya *et al*. 2017 emend. Kallscheuer *et al*. 2025, 9*	emend.	[22]
*Tundrisphaera lichenicola* Kulichevskaya *et al*. 2017 emend. Kallscheuer *et al*. 2025, 9*	emend.	[22]
*Xanthomonas* Dowson 1939 (Approved Lists 1980) emend. Bansal *et al*. 2023, 462	emend.	[10]
*Xanthomonas maltophilia* (Hugh 1981 *ex* Hugh and Ryschenkow 1961) Swings *et al*. 1983 emend. Bansal *et al*. 2023, 462	emend.	[10]

*The journal in which the name was effectively published does not have continuous pages.

†The authors gave *Alicyclobacillus tolerans* K1.

‡The authors gave Lee *et al.* 2023 instead of Lee *et al.* 2025.

§The authors gave Xu* et al.* 2020 instead of Feng *et al.* 2015.

¶The authors incorrectly stated that the type species of the genus *Thermoanaerobacter *is now *Thermoanaerobacter thermohydrosulfuricus*, as *Thermoanaerobacter ethanolicus *subsp. *ethanolicus *was proposed as a heterotypic synonym of *T. thermohydrosulfuricus*.

For references to Lists of Changes in Taxonomic Opinion nos. 1–43, see *Int J Syst Evol Microbiol* **55** (2005) 7, 1403; **56** (2006) 11, 1463; **57** (2007) 4, 1376; **58** (2008) 5, 1515; **59** (2009) 5, 1559; **60** (2010) 5, 1482; **61** (2011) 4, 1504; **62** (2012) 7, 1448; **63** (2013), 8, 2371, **64** (2014), 8, 2191; **65** (2015), 7, 2028; **66** (2016), 7, 2469; **67** (2017), 7, 2081; **68** (2018), 7, 2137; **69** (2019), 13, 1850; **70** (2020), 9, 4061; **71** (2021), 004596, 004816; **72** (2022), 005164, 005413; **73** (2023), 005696, 005923; **74** (2024), 006145, 006482; **75** (2025), 006561, 006793; **76** (2026), 006976.

## Abbreviations

ICNP: International Code of Nomenclature of Prokaryotes
IJSEM: International Journal of Systematic and Evolutionary Microbiology
